# Neuroimaging features of xeroderma pigmentosum group A

**DOI:** 10.1002/brb3.22

**Published:** 2012-01

**Authors:** Takehiro Ueda, Fumio Kanda, Nobukazu Aoyama, Masahiko Fujii, Chikako Nishigori, Tatsushi Toda

**Affiliations:** 1 Division of Neurology, Kobe University Graduate School of MedicineKobe, Japan; 2 Division of Radiology, Kobe University Graduate School of MedicineKobe, Japan; 3 Division of Dermatology, Kobe University Graduate School of MedicineKobe, Japan

**Keywords:** Brain MRI, Central nervous system, Diffusion tensor imaging, Fractional anisotropy, Magnetic resonance spectroscopy, Xeroderma pigmentosum

## Abstract

Xeroderma pigmentosum group A (XPA) is a hereditary dermatological disease in which hypersensitivity to ultraviolet radiation and various neurological symptoms are observed. In this study, to evaluate the degeneration occurring in the brain of XPA patients, neurological examinations by an established neurologist and 3-Tesla magnetic resonance imaging (MRI) were performed in 10 Japanese XPA patients. MRI studies included diffusion tensor imaging (DTI) and magnetic resonance spectroscopy (MRS) in addition to conventional sequences. Neurological examinations revealed various deteriorations in the both central and peripheral nervous systems in all subjects. MRI studies demonstrated age-dependent decline in multimodalities. Severe brain atrophy in conventional sequences, decreased fractional anisotropy (FA) value in DTI, and reduced NAA/Cre ratio in MRS were observed in the adult patients. Multimodal MRI studies unmask the neurological deterioration in XPA patients.

## Introduction

Xeroderma pigmentosum (XP) is a congenital autosomal recessive disease in which photosensitivity and skin cancer due to sun exposure are observed. Eight complementation groups have been described in XP. Groups A–G (XPA–XPG) show defects in nucleotide excision repair of deoxyribonucleic acid (DNA), while the XP variant (XPV) shows a defect in replication of DNA templates carrying unrepaired DNA damage. In XPA, various neurological symptoms are observed apart from dermatological problems (Mimaki et al. 1986). In recent years, the remarkable progresses in avoidance of sun exposure and in treatment for skin cancer have markedly improved the life expectancy of XPA, thereby the neurological disorders have emerged as the critical factors that affect daily life and disease prognosis (Anttinen et al. 2008).

Relentless progression of neurological deterioration continues even in XPA patients who avoid sun exposure. Although atypical cases with mild neurological complications have been reported (Robbins et al. 1991; Anttinen et al. 2008), the most of XPA patients follow a similar clinical course in which they gradually deteriorate from having neurological symptoms in childhood to being bedridden in adulthood (Robbins et al. 1991). The pathogenesis of neuronal injury in XPA is still unclear and there are no treatments available. Pathological studies on autopsy brain were performed in a few XPA patients who reached adulthood (Kanda et al. 1990; Itoh et al. 1999), and revealed extensive loss of neurons and gliosis of the white matter in the central nervous system (CNS). Only few studies evaluated CNS involvement of XPA patients using head computed tomography, electroencephalography, or cognitive function testing (Mimaki et al. 1989; Robbins et al. 1991; Anttinen et al. 2008). There have been no reports on detailed magnetic resonance imaging (MRI) analysis of pediatric XPA patients.

In this preliminary study, we analyzed brain disorders in XPA patients using several MRI sequences.

## Subjects and Methods

Ten genetically proven Japanese XPA patients were studied (Table 1). All patients had history of severe sunburn at the first sun exposure after birth and were diagnosed on the basis of the clinical episode and measurement of the minimal erythema dose. Most patients, except for No.7 and No.10, were genetically determined as having mutation c.390–1G>C in *XPA* by polymerase chain reaction restriction fragment length polymorphism using restriction enzyme AlwNI according to a previously described method (Nishigori et al. 1994). Each patient underwent neurological examination by an established neurologist and imaging studies on the same day. Images were obtained using a whole-body 3-Tesla MRI system (Phillips Medical Systems, Eindhoven, The Netherlands).

**Table 1:** Neurological examinations and 3-Tesla MRI results in 10 Japanese XPA patients

Patient no.	Sex	Age	DTRs	Babinski's sign	Ataxia	Rigidity	Hearing loss	ADL
1	M	1	dim.	ext.	−	−	−	Stand holding on to something
2	M	1	dim.	flex.	−	−	−	Swaying walk
3	M	6	dim.	ext.	+	−	+	Walk unaided
4	F	6	dim.	ext.	+	−	+	Walk unaided
5	M	7	abs.	ext.	+	−	+	Walk unaided
6	F	8	dim.	ext.	+	−	−	walk unaided
7	F	8	abs.	ext.	+	−	+	Walk unaided
8	F	9	abs.	ext.	+	−	−	Walk unaided
9	M	18	abs.	ext.	+	+	+	Wheelchair
10	F	20	abs.	ind.	−	+	+	Bedridden

M = male, F = female, DTRs = deep tendon reflexes (dim. = diminished, abs. = absent), Babinski's sign = abnormal plantar reflex (ext. = extensor, flex. = flexor, ind. = indifferent), ADL = activity of daily living. In ataxia, rigidity, and hearing loss, “+” means present and “−” means absent.

At first, we performed conventional sequences including T1-weighted images (T1WI) (echo time (TE) = 3.3 msec, repetition time (TR) = 7.2 msec, flip angle = 8°, field of view (FOV) = 256 × 256 mm^2^, matrix = 512 × 512, slice thickness (ST) = 0.8 mm), T2-weighted images (T2WI) (TE = 120 msec, TR = 3500 msec, flip angle = 90°, FOV = 230 × 230 mm^2^, matrix = 512 × 512, ST = 5.0 mm), and fluid-attenuated inversion recovery (FLAIR) imaging (TE = 125 msec, TR = 11000 msec, flip angle = 90°, FOV = 230 × 230 mm^2^, matrix = 512 × 512, ST = 5.0 mm).

**Figure 1:**
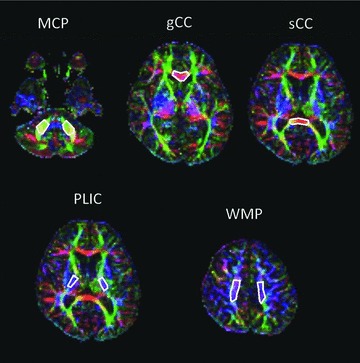
Regions of interest (ROI) on the fractional anisotropy (FA) color map are shown. MCP = middle cerebellar peduncle, gCC = genu of corpus callosum, sCC = splenium of corpus callosum, PLIC = posterior limb of the internal capsule, WMP = white matter of the parietal lobe.

Next, we performed diffusion tensor imaging (DTI) (TE = 80 msec, TR = 8052 msec, flip angle = 90°, FOV = 256 × 256 mm^2^, matrix = 128 × 128, ST = 2.0 mm, motion probing gradient = 15 directions, *b*-value = 1000) and created fractional anisotropy (FA) maps using DTI Studio software (http://cmrm.med.jhmi.edu/). Regions of interest (ROI) were set in four locations on the basis of the DTI color map atlas (Wakana et al. 2004) (Fig. 1): the middle cerebellar peduncle (MCP, 30 voxels; bilateral 15 voxels each), posterior limb of the internal capsule (PLIC, 14 voxels; bilateral seven voxels each), corpus callosum (CC, 40 voxels; splenium and genu 20 voxels each), and white matter of the parietal lobe (WMP, 30 voxels; bilateral 15 voxels). We calculated mean FA values of 10 times settings in each location except when the standard deviation was higher than 0.1.

**Figure 2:**
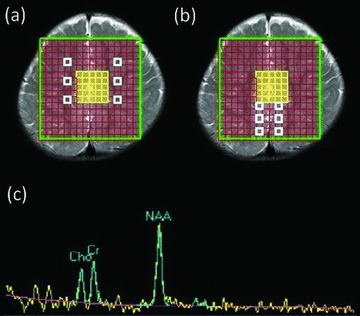
In magnetic resonance spectroscopy (MRS) study, six voxels (three voxels on each side) in centrum semiovale (A = white boxes) and gyrus cinguli (B = white boxes) as region of interest. Spectrum of NAA, creatine, or choline is shown (C).

In addition, we performed multivoxel magnetic resonance spectroscopy (MRS) (TE = 144 msec, TR = 2000 msec, FOV = 80 × 80 mm^2^, voxel of interest (VOI) size = 65 × 65 mm^2^, matrix = 16 × 16, ST = 15 mm, voxel resolution = 5 × 5 × 15 mm^3^). Multivoxels were set on the section containing gyrus cinguli and centrum semiovale (frontoparietal white matter site) along with a reference (Doelken et al. 2009). As the parameter, N-acetyl aspartate (NAA), creatine (Cre), choline (Cho), lactate, and lipid were analyzed. We chose six voxels (three voxels on each side) in gyrus cinguli and centrum semiovale as ROI and calculated the mean NAA/Cre ratio and the mean Cho/Cre ratio in these regions (Fig. 2).

In accordance with the ethical guidelines of the Declaration of Helsinki, written informed consent was obtained from all participants’ guardians under protocols approved by the Institutional Review Board of Kobe University Graduate School of Medicine, Kobe, Japan.

## Results

### Neurological examinations

Patient profiles and neurological findings are summarized in Table 1. Hearing loss is a common symptom in XP. Progressive intellectual deterioration disturbed accurate evaluation for sensation. Deep tendon reflexes (DTRs) were absent or diminished in all patients, and abnormal plantar reflex (Babinski sign) were positive in eight of 10 patients. Those neurological findings indicated that both upper and lower motor neuron involvement started from an early stage. Cerebellar ataxia was obvious after 6 years of age. In an 18-year-old patient, an extrapyramidal sign was observed in association with various neurological abnormalities. In patient No.10, severe contractures in all extremities resulted in impossible proper examination for the presence of ataxia or abnormal reflexes.

### Conventional MRI sequences

**Figure 3:**
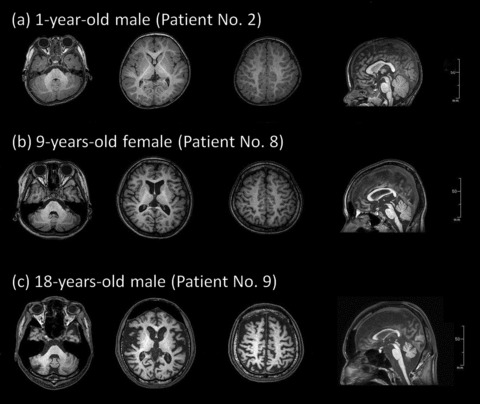
Three axial views and one sagittal view from T1-weighted MRI images in (A) 1-year-old, (B) 9-year-old, and (C) 18-year-old XPA patients.

T1WI showed that diffuse brain atrophy was severe in elderly XPA patients (Fig. 3). T2WI and FLAIR imaging showed no abnormality such as vascular disease or metal deposits in any patient. The arterial flow void was also intact (figures not shown).

### Diffusion tensor imaging (DTI)

**Figure 4:**
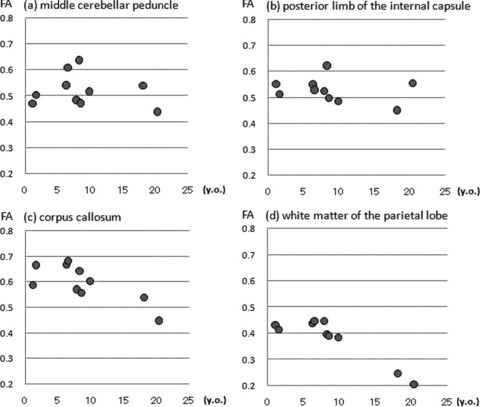
FA values in XPA patients are plotted against the age of patients.

We calculated mean FA values and plotted the results against the age of patients (Fig. 4). For most XPA patients between 6 and 9 years of age, FA values at any region differed little from those in the 1-year-old patient. Furthermore, FA values were lower in patients No.9 and No.10 compared with younger patients, especially in the CC and WMP.

### Magnetic resonance spectroscopy (MRS)

**Figure 5:**
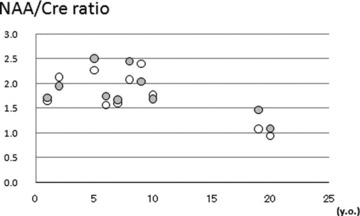
NAA/Cre ratios in the centrum semiovale (open circles) and gyrus cinguli (filled circles) in XPA patients are plotted against the age of patients.

Peaks for lactate and lipid were not detected in any patient. The NAA/Cre ratios were plotted against the patients’ age shown in Figure 5. The NAA/Cre ratios ranged from 1.5 to 2.5 for patients No.1 through No.8 but fell below 1.5 for patients No.9 and No.10. Cho/Cre ratios had no differences between all patients (data not shown).

## Discussion

The molecular mechanism for neuronal damage in XPA is yet to be elucidated. Recently, it has been discussed that acquired factors such as oxidative stress or excitatory amino acid toxicity are related to CNS disorders in XPA. It was reported that metabolic products of oxidative stress were exhibited in the basal ganglia in the brain of XPA patients, while apoptosis, neurofibrillary tangles, or senile plaques were not noted (Hayashi et al. 2005). Due to the inability to repair DNA in XPA patients, acquired damage could be a factor in the neurodegenerative changes. DNA damage from oxidative stress, however, is commonly corrected by “base” excision repair (Robertson et al. 2009). Oppositely, XPA is a disorder of “nucleotide” excision repair system. Unknown mechanism, other than malfunction in DNA repair, is assumed to play an important role in neuronal damage in XPA.

Neurological symptoms are common in XPA patients, though its precise mechanism remains still unclear. The onset of neurological symptoms in XPA seems to occur between 3 and 8 years of age (Anttinen et al. 2008). In our study, however, even 1-year-old patients showed neurological abnormalities such as a decline of DTRs. Some patients had history of several months delay of initial walking. Contrary to general understanding, careful observation can detect neurological symptoms in infancy in XPA patients.

Conventional MRI sequences showed brain atrophy and expansion of frontal sinuses in adolescent patients. Every region of the brain, including cortex, brain stem, and cerebellum, remarkably reduced in size in adult patients. There was no diagnostic focal atrophy, such as spinocerebellar atrophy or corticobasal degeneration. Both white matter and gray matter were reduced similar in severity.

In order to detect the structural change in white matter, we measured FA values in several regions of the brain in XPA using DTI. In normal children, the FA values of the brain increases until 2 or 3 years of age and remained approximately constant thereafter (Hermoye et al. 2006). Other reported that FA values were almost the same from 5 to 20 years of age in normal controls (Schneider et al. 2004). After that, FA values tended to gradually decline after 20 years of age (Moseley 2002). In our study, FA values were almost the same in any age in child XPA patients, and were appeared to decline in adolescent patients. Lack of increase in FA in infancy might indicate that some damages of the CNS start very early in the life of XPA patients. Those early reductions in FA in the brain of XPA are consistent with those in other congenital developmental disorders such as Prader–Willi syndrome (Yamada et al. 2006) or autism spectrum disorder (Shukla et al. 2011).

Furthermore, we used MRS to assess metabolic function of the brain of XPA patients. In the adult patients (No. 9 and No. 10), the NAA/Cre ratio was lower in both the cerebral cortex (gyrus cinguli) and white matter (frontoparietal region) than those in the other child patients. Reduction in NAA/Cre represents nonspecific neuronal damage. We did not find any specific metabolic abnormalities in the brain of XPA.

In our study, delicate neurological examination and multimodal MRI studies unmask the onset of neurological deterioration in XPA patients in early stage. Further accumulative and longitudinal studies are needed.
